# The influence of ensemble size and composition on the performance of combined real-time COVID-19 forecasts

**DOI:** 10.1371/journal.pcbi.1014908

**Published:** 2026-11-03

**Authors:** Friederike Becker, Katharine Sherratt, Nikos I. Bosse, Sebastian Funk

**Affiliations:** 1 Institute of Statistics, Karlsruhe Institute of Technology, Karlsruhe, Germany; 2 Centre for Mathematical Modelling of Infectious Diseases, London School of Hygiene & Tropical Medicine, London, United Kingdom; 3 Department of Infectious Disease Epidemiology and Dynamics, London School of Hygiene & Tropical Medicine, London, United Kingdom; The University of Melbourne, AUSTRALIA

## Abstract

During infectious disease outbreaks, short-term forecasts can play an important role for both decision makers and the general public. While previous research has shown that combining individual forecasts into an ensemble improves accuracy and consistency, practical guidance for organisers of multi-model prediction platforms on how to construct an ensemble has been scarce. In particular, it is not entirely clear how ensemble performance relates to the size of the underlying model pool, a relevant question when relying on voluntary contributions from modelling teams that face competing priorities. Furthermore, the exact composition of an ensemble forecast may influence its performance. Ensembles can either include all models equally or, alternatively, discriminate based on past performance or other characteristics. Using data from the European COVID-19 Forecast Hub we investigated these questions, with the aim of offering practical guidance to organisers of multi-model prediction platforms during infectious disease outbreaks. We found that including more models both improved and stabilised aggregate ensemble performance, while selecting for better component models did not yield any particular advantage. Diversity among models, whether measured numerically or qualitatively, did not have a clear impact on ensemble performance. These results suggest that for those soliciting contributions to collaborative ensembles there are more obvious gains to be made from increasing participation to moderate levels than from optimising component models.

## Author summary

Infectious disease outbreaks such as the recent COVID-19 pandemic present uncertain situations for governments, health agencies and the general public. Short-term forecasts of key indicators, such as case numbers and deaths from the disease, can help inform expectations about how an outbreak is likely to develop in the near future. These forecasts are often produced using computer models that differ in their underlying assumptions and use of available data. One common approach to improve prediction quality is to collect forecasts from several such models and combine them into a so-called ensemble forecast. However, for organisers of these forecasting efforts it is often unclear how to best construct such an ensemble, in particular how many individual forecasts to collect or whether some models’ forecasts should be selected over others. Using forecasts submitted to the European COVID-19 Forecast Hub, we examined how ensemble performance depends on the number and selection of contributing model forecasts. We found that ensembles generally became more accurate and more stable as additional models’ forecasts were included. In contrast, selectively including only models with better past performance did not consistently improve results. We also found no clear evidence that greater diversity among modelling approaches led to better ensemble forecasts. Our findings suggest that ensemble forecasting efforts benefit more from encouraging broad participation to obtain a moderate number of forecasts than from selecting individual models’ forecasts.

## Introduction

Epidemic forecasting can be a useful tool during infectious disease outbreaks, reducing short-term uncertainty and supporting planning and resource allocation [1]. Repeated studies have demonstrated increased forecast accuracy from the combination of multiple model predictions into an ensemble. This result has been noted since the 1960s [2], and repeatedly observed in outbreak forecasting across multiple pathogens and outbreaks over time [3–7]. It also motivates calls for continued investment in large-scale modelling collaborations producing ensemble forecasts [8].

Meanwhile, collaborative ensembles are typically “ensembles of opportunity” [9], combining across a non-systematically selected, and potentially fluctuating, sample of contributing models with limited treatment of model interdependence or performance in ensemble construction. Therefore, even while ensemble predictions tend to outperform the majority of individual models, it is important to assess whether this performance is robust to different compositions of contributing models. Evaluating contributing factors for creating ensemble outperformance can also support the design of future collaborative modelling projects.

Contributing factors to ensemble performance may be the number of member component models, the diversity of those models, and their individual predictive performance. Previous work analysing US influenza and COVID-19 forecasts has characterised the impact of the number of component models [10]. This found that among a random selection of models, including more models improved ensemble performance, particularly in the range of up to 4 models, while also decreasing the variability of ensemble performance.

Studies in various fields have discussed and empirically shown the value of including a diverse set of forecasts in an ensemble [11,12]. Past work on forecasting infectious diseases has investigated differences in performance across methodological types at the individual model level [3,13], but has not identified the impact of combining across these types on ensemble performance. It thus remains unclear whether diversely composed ensembles outperform those of a single modelling type, a question we address directly.

Apart from diversity, an ensemble might identify or weight its potential members based on their individual performance. In many applications, equally weighted ensembles are not outperformed by approaches that assign model weights based on individual past performance [14]. In related analyses to ours, Bracher et al. [15] found no systematic benefits of a weighted ensemble using data from Germany and Poland, while Ray et al. [16] have conversely found a weighted approach to be beneficial in forecasting COVID-19 Deaths (but not Cases), based on data from the US.

In this work we explore the impact of these factors, that is, ensemble size, component model diversity, and individual model performance, in the context of the European COVID-19 Forecast Hub, a collaborative multi-model project set up in 2021 to inform the response to the COVID-19 pandemic across Europe. We approach this from two complementary angles. First, we systematically vary ensemble size and composition through a recombination procedure, investigating how performance depends on the number and diversity of contributing models when these are sampled from the available pool. Second, we investigate if ensembles that actively select for recent better-performing models can improve upon an equally weighted aggregation of all available models. Together, these analyses examine whether ensemble performance is driven more by the size of the contributing pool or by its composition in terms of model diversity and individual performance.

## Methods

### Scope

We used forecast data from the European COVID-19 Forecast Hub [17], a coordinated and collaborative forecasting effort instigated by the European Centre for Disease Prevention and Control (ECDC) in 2021. The Hub collated weekly forecasts from independent modelling teams for COVID-19 Deaths and Cases, for 1–4 weeks into the future and across 32 European countries. Forecasts were submitted in a probabilistic and standardised format, via 23 predictive quantiles, that is, 11 central prediction intervals plus a median forecast.

All forecasts were evaluated against ground truth data collated for public use by Johns Hopkins University (JHU), with weekly incident Cases and Deaths derived from the daily cumulative counts reported by JHU by taking differences, as was done in the data set used as ground truth for the Hub.

During the period studied here, the Hub included a naive baseline model (last value carried forward, with uncertainty stemming from previous weekly differences of the series, analogous to [6]) and an official Hub ensemble, which aggregated all eligible component forecasts initially via the mean and, later, the median function, and was used by the ECDC in communication about the development of the pandemic.

In our analysis, we used a “Hub-replica” ensemble as a benchmark for all comparisons. This ensemble aggregated all eligible models via the median function at all forecast dates, thus removing the structural break that the switch of aggregation method presented in the official Hub ensemble data.

Modelling teams were free to choose which combinations of location and target series they submitted forecasts for, leading to substantial variation in model availability across location-target combinations. Since the set of contributing models differed across these combinations, we conducted all analyses separately by location and target.

Throughout our analyses, we constructed alternative ensembles, either through performance-based selection or exhaustive recombination, to investigate the impact of changing the composition of the ensemble forecast. For this, we relied critically on the size of the available model pool to obtain sufficient sample sizes. We thus limited ourselves to a subset of the Hub data that generally saw higher numbers of individual submissions from the independent modelling teams, while still retaining some generality. Specifically, we used forecasts that were issued for Germany, Poland, the Czech Republic, the United Kingdom and France, for both Deaths and Cases, between mid-March 2021 and mid-March 2022. This gave us 52 forecast dates in total. Fig 1 displays the number of models that were available for these locations and both target series. Submission levels were higher during the summer and fall months of 2021, and the highest levels of participation were generally observed in Germany and Poland. The temporal pattern of model availability alongside epidemic incidence is further illustrated in Fig A in S1 Text.

**Fig 1:**
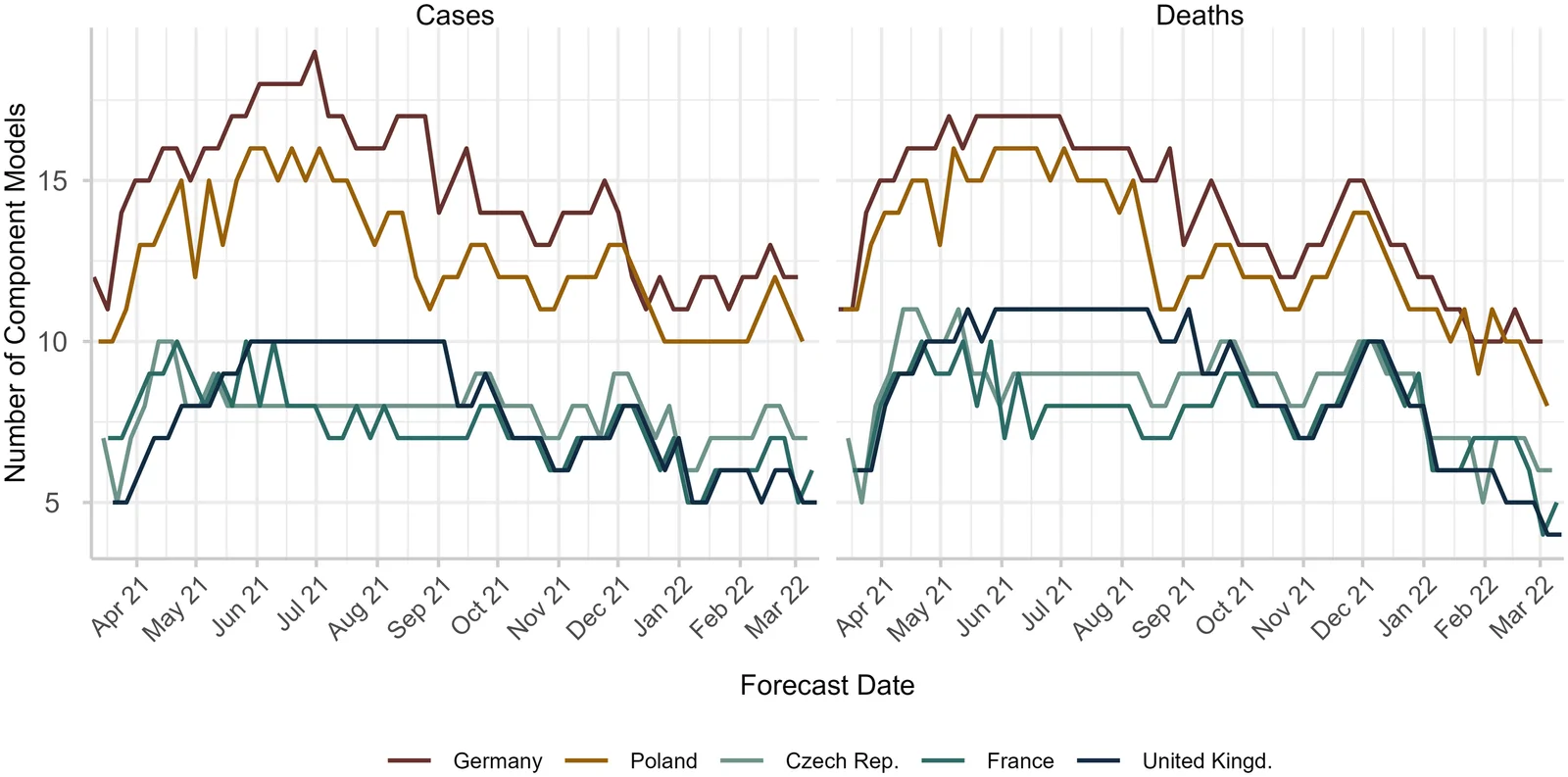
Number of available component models for each location and the two target series, COVID-19 Cases and Deaths.

For our study, we only considered “full” forecasts, that is, those that were made for all horizons up to 4 weeks ahead and all 23 quantile levels. This choice is consistent with the Hub’s own eligibility criteria for inclusion in the official ensemble [18]. We generally focused on communicating results for the 1- and 2-week horizons. This choice is due to conciseness, and where appropriate, we refer to results for all horizons in S1 Text.

### Construction of ensembles of different size

For the first part of our analysis, we constructed ensembles by recombining models from the available model pool, which is specific to each combination of target series *j* (Cases and Deaths) and forecast location (country) *i*. For each location-target, and for each *k*, we thereby obtained (Kijk) proposed ensembles, where $K_{ij}$ is the total number of models that have at some point submitted a forecast for target *j* and location *i*.

The available forecast data was characterised by intermittent submission (not all models were submitted for all dates, see variability in available models in Fig 1), which posed some issues for scoring forecasts, see section “Forecast evaluation” for more detail. We thus imposed some availability thresholds on individual models and the recombined ensembles. Individual models were considered as eligible for recombination if forecasts were available for at least half of the study period. We thus obtained (K~ijk) suggested recombined ensembles, each of which was then considered eligible to be scored as an alternative ensemble if all models were *jointly* available for at least half of the study period. The final ensembles were then aggregated with the median function. The procedure is illustrated in the top panel of Fig B in S1 Text.

Recombined ensembles were additionally classified as being either “homogeneous” (all included component models were of the same general modelling type, either “mechanistic”, “semi-mechanistic” or “statistical”) or “heterogeneous” (included component models were of 2 or more different types). Component models were classified based on metadata provided by the teams, following the same classification as in [19], which provides further details on the process. Using this classification of ensembles we could investigate whether those ensembles that incorporated diverse modelling approaches tended to perform better than those that relied on a single model type.

### Construction of selection ensembles

For the second part of our analysis, we investigated if and how it may be beneficial to compose an ensemble by selecting for better recent performers, possibly in conjunction with additionally weighting models based on their recent performance.

To this end, we used a rolling window approach at each forecast date to select the *k* best recent performers, based on 1- and 2-week horizon scores for the respective location and target series in the past 4 weeks. More details on scoring are given in section “Forecast evaluation”. To best reflect the original real-time setting, we only included forecasts that had already realised at the respective forecast date, thereby excluding the most recent 2-week forecast. For the number of models selected into the ensemble, we considered *k* = 3,5,8,10. The *k* chosen models were then median-aggregated with two methods, once equally and once via their inverse score in the past 4 weeks - as scores are negatively oriented (lower values are better), better recent performers thus received a higher weight. The procedure is illustrated in the bottom panel of Fig B in S1 Text.

While the Hub favoured the median due to its robustness to potential outlying forecasts, we additionally investigated mean aggregation here, again with both equal and inverse-score weights. It is possible that the mean could perform better in the given setting, where we selected for better recent performers and thus potentially excluded those models that might be prone to outlying predictions.

Given that our analysis was entirely retrospective, we refrained from excessively tuning the window size to the point where it would give the most favourable in-sample results. We therefore chose a similar value as [15], who used a 3-week window in a related analysis, and slightly increased this to 4 weeks because we had access to a larger dataset.

Sherratt et al. [16] and Ray et al. [7] conducted similar analyses on the European Hub data. However, the former did not treat locations separately and thus did not account for the considerable difference in model availability by location, while the latter did not preselect models before weighting them.

### Forecast evaluation

Similar to previous analyses on the US and European COVID-19 Forecast Hubs [6,7], we scored all forecasts with the weighted interval score (WIS) [20], a proper scoring rule designed for forecasts that are submitted in an interval format. The WIS is negatively oriented, that is, smaller values correspond to better forecasts. It evaluates both the calibration of forecast intervals (e.g., whether a nominal 80% forecast interval covers close to 80% of observations over time) and their sharpness, that is, how informative they are.

In the first part of our analysis (see section “Construction of ensembles of different size”), different ensembles were not generally available on the same forecast dates. Because absolute WIS values tend to be lower during low-incidence periods, ensembles that happened to be evaluated predominantly during such periods would receive lower average scores. We addressed this issue by scoring with pairwise comparisons [6]. Briefly, this procedure computes relative skill for each pair of ensembles as the ratio of their average scores on shared forecast dates only, thus avoiding the bias introduced by incomplete overlap in availability. Each ensemble’s total relative score is then computed as the geometric mean of relative skill values across all comparators, and subsequently scaled against the Hub-replica’s relative score. We thereby obtained a concise measure of performance, scaled relative skill, which can be interpreted as follows: a value > 1 means that the respective alternative ensemble performed worse than the Hub-replica ensemble, while a value < 1 means that it performed better.

In the second part of our analysis, we constructed ensembles that selected for better performers on a rolling basis. The resulting ensembles were available for all forecast dates and we thus obtained a comparable measure of performance by dividing the WIS of the selection ensemble by that attained by the Hub-replica ensemble. Again, a value > 1 means that the respective alternative ensemble performed worse than the Hub-replica ensemble, and vice versa.

### Computation

All analyses were conducted using the statistical software R. The code for this analysis can be found here: https://github.com/epiforecasts/eurohub-ensembles. All scoring and performance evaluation was done with the scoringutils R package [21].

### Use of AI tools

During the revision of this work, we used an AI coding assistant (Claude Code, based on Claude Opus, Anthropic) to review the analysis code for correctness and reproducibility and to help implement the resulting fixes. Furthermore, we used Claude to refine the wording of specific text passages, to assist with two supplementary figures (Figs A and J in S1 Text) and to check the manuscript for consistency and errors. All code changes and text edits were reviewed, tested, and verified by the authors, who take full responsibility for the analyses and results reported here. All results, interpretations, and conclusions represent the authors’ own ideas.

## Results

### Ensemble size and diversity

Scaled relative skill values for the recombined ensembles are shown in Fig 2, respectively for Cases and Deaths and the two forecast horizons. For each set of recombined ensembles of size *k*, we show the median scaled relative skill, as well as the min-max and 5%−95% quantile range.

**Fig 2:**
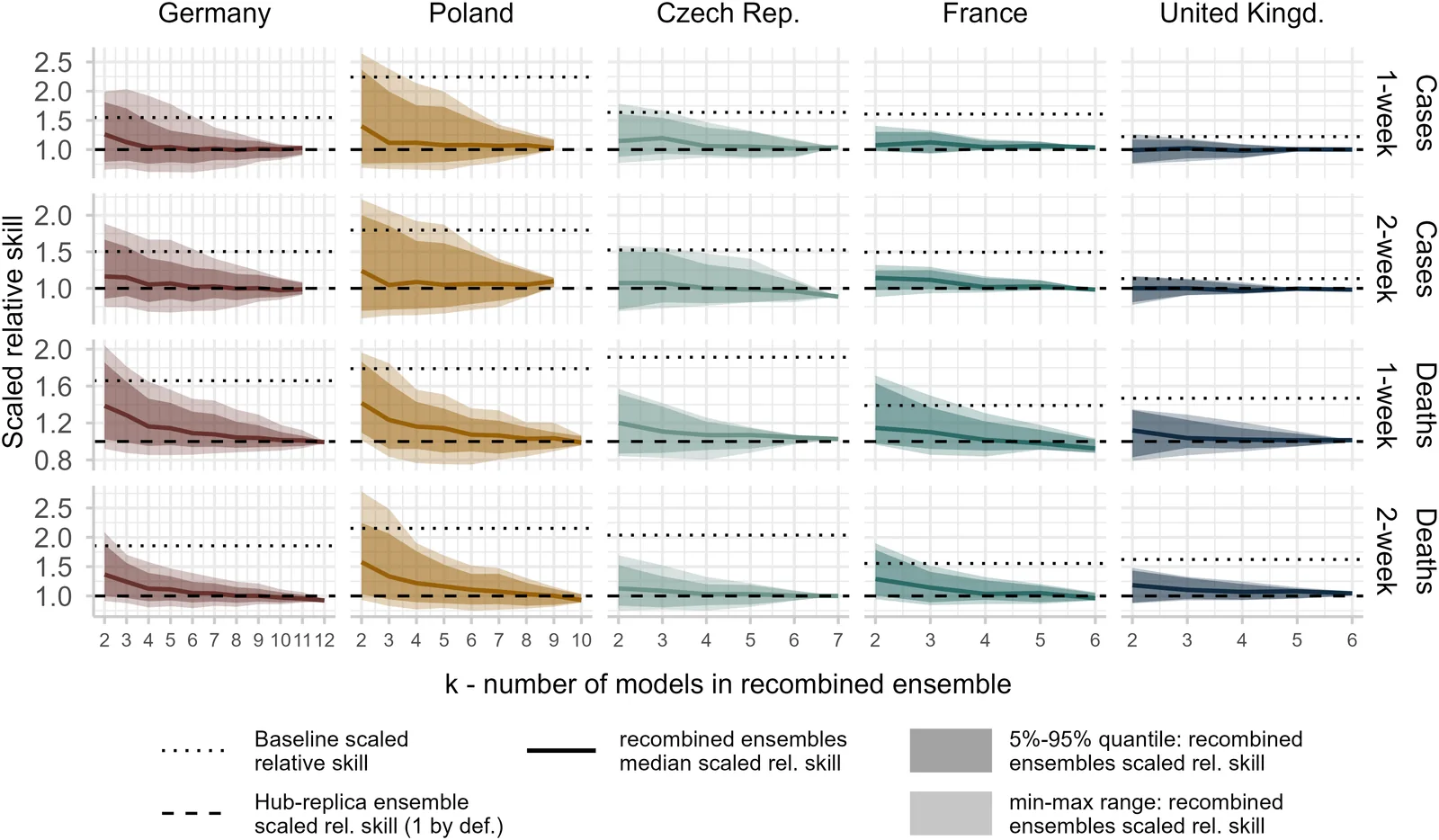
Ensemble recombination results for COVID-19 Cases and Deaths, using data from the European COVID-19 Forecast Hub. Models were recombined from the full set of available forecast models to create alternative ensembles of size *k*, which were scored via the weighted interval score and pairwise comparisons, and subsequently scaled against the performance of the full Hub-replica ensemble. Recombined ensembles were aggregated with the median function. Curves differ by length, depending on the size of the available underlying model pool.

For both target series and all horizon-location combinations, the benefits of ensembling component forecasts were apparent, with values of scaled relative skill generally decreasing with a larger number of component models in the recombined ensembles. With few exceptions, recombined ensembles with 4 members or more outperformed the naive baseline model. For most series, the largest decreases in median scaled relative skill tended to occur between 2 and 4 component models, after which performance typically either plateaued or improved more slowly. In particular, larger digit percentage decreases in median scaled relative skill across all recombined ensembles were typically limited to smaller values of *k*, see Fig C in S1 Text, while percentage changes for larger *k* tended to stay closer to 0. In some instances, moderate improvements continued to higher values of *k*, e.g., for Poland Deaths.

Spread in relative performance also considerably decreased with *k*, suggesting that including more models may remove the need to select component models that are particularly beneficial for ensemble performance. Some of this effect is likely attributable to the recombined ensembles simply becoming more similar in their composition. However, the decrease in performance variability was still apparent in the range of smaller *k* values, where there were still recombined ensembles that had no overlap in the models that they included.

Overall, differences between the forecast horizons and the target series appeared small, with all curves largely following the same pattern. Between the different locations, the number of available component models was a major factor in the different forms of the curves.

Additionally, Fig 3 shows the range in scaled relative skill for recombined ensembles whose component members are all of the same model type (“homogeneous”) or of 2 or more different model types (“heterogeneous”). While sample sizes were small for homogeneous ensembles in particular, the performance ranges for the groups were similar, and in particular not clearly in favour of heterogeneous ensembles, especially for ensembles with more than 2 members. When considering a direct numerical measure of distance between component model predictions in each recombined ensemble, see Fig D in S1 Text, there also appeared to be no clear relationship between diversity in an ensemble and that ensemble’s performance.

**Fig 3:**
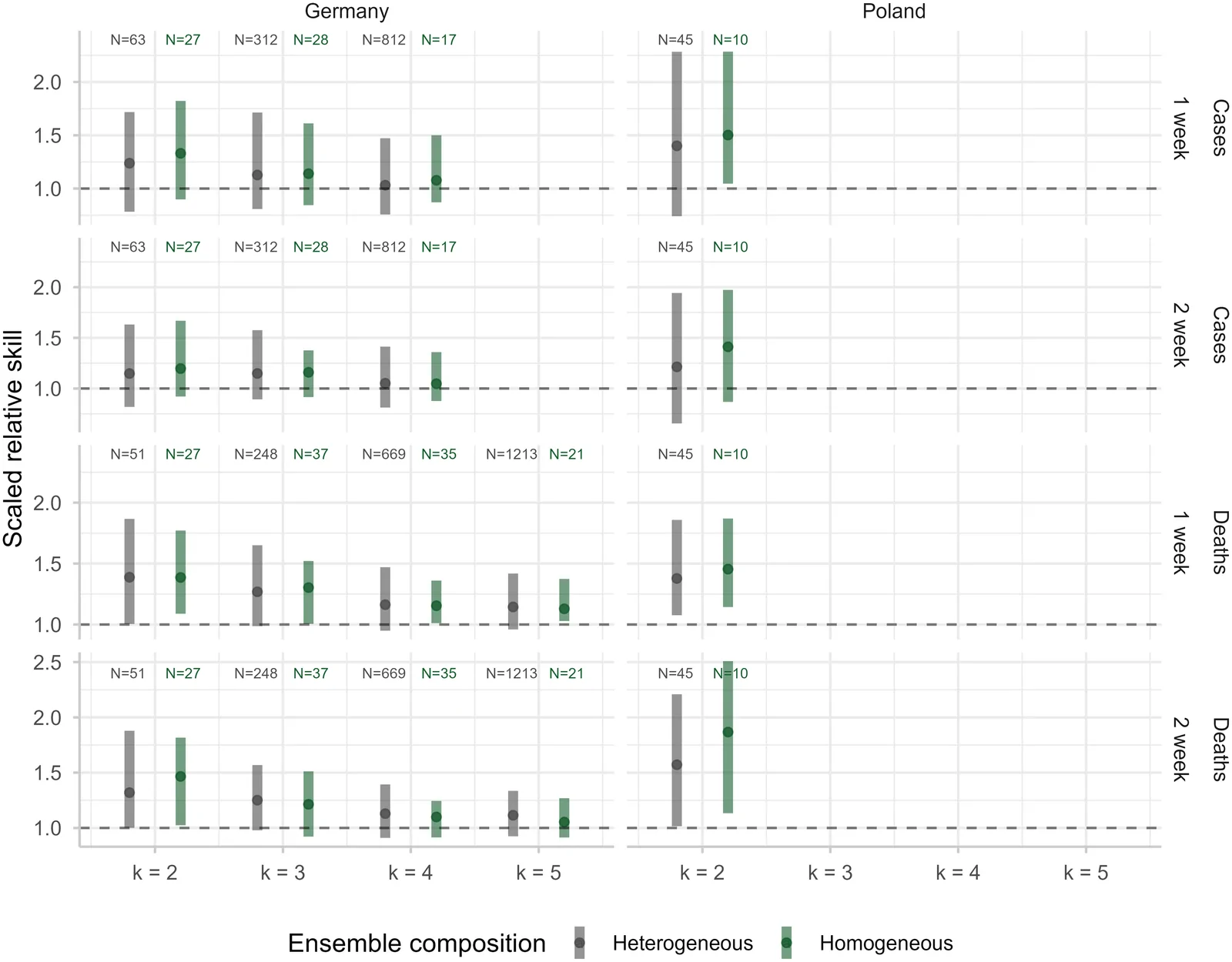
Same data as Fig 2, with ensembles split up by types “homogeneous” (all component models in the recombined ensemble were of the same model type: either “mechanistic”, “semi-mechanistic”, or “statistical”) and “heterogeneous” (component models in the ensemble were of 2 or more different model types). Shown are 5% to 95% quantile ranges for scaled relative skill (shaded areas) and median scaled relative skill (points). Data is only shown for combinations of location, target series and *k* for which there were at least 10 recombined ensembles of each type. The number of available recombined ensembles is indicated at the top of each plot, via “N”.

The kinks in the relative skill curves in Fig 2 could be attributed to the median aggregation method, which repeatedly alternated between selecting the midpoint prediction (odd number of component models) and averaging between the two midpoint predictions (even number of component models). Particularly in the range of smaller *k*, this did have an effect on results. Fig E in S1 Text shows the results from the same experiment with mean aggregation. Overall, the results were very similar, with the curves following the same patterns but appearing noticeably smoother.

### Selection ensemble

While the recombination experiment showed that more models generally improved and stabilised performance, it left open whether a full ensemble could be outperformed by deliberately choosing models that performed well in the recent past, which we investigate next.

Fig 4 shows the average WIS values of the selection ensembles, relative to the Hub-replica benchmark ensemble. Especially when averaged across all locations (leftmost column in both plots), relative WIS values stayed close to 1 in most cases, indicating no substantial performance gains (or losses) when preselecting for recent better performers, whether or not they were subsequently weighted.

**Fig 4:**
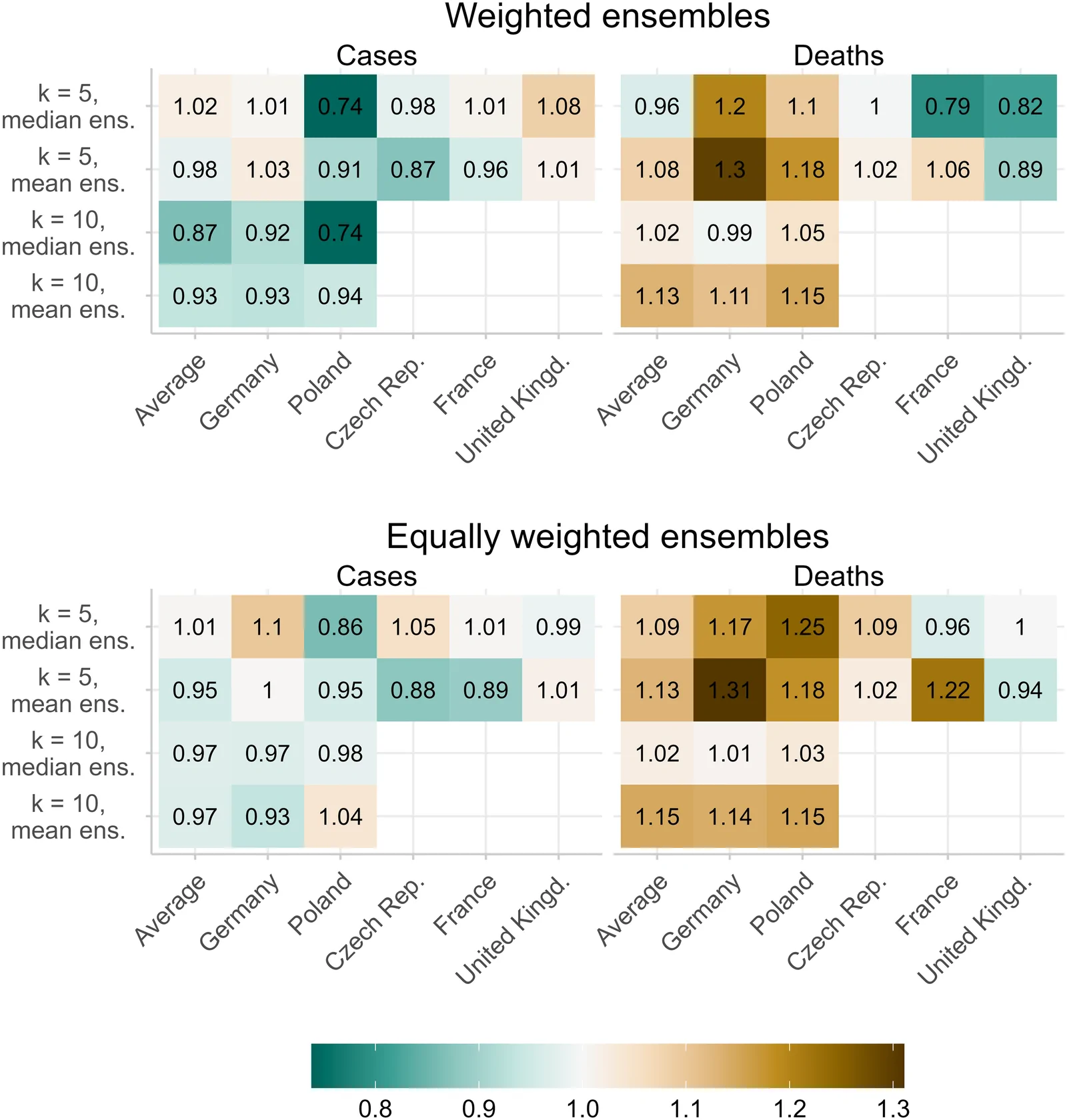
Relative WIS of the (weighted) selection methods, compared to the full Hub-replica ensemble, averaged for the 1- and 2-week forecast horizons. The selection method reduced the set of component forecasters to the number of *k* best recent performers - different aggregation methods were then applied to obtain the ensemble (weighted or unweighted mean or median). The “Average” location refers to the setting where scores were averaged across all locations. Relative scores are missing whenever a location’s model pool was not large enough to support the analysis.

In fact, for any given aggregation method, the selection ensemble tended to both outperform and be outperformed by the benchmark in some locations, suggesting that sample variation might be a key factor underlying the relative performance scores. The two target series differed in how much the selection procedure altered performance with respect to the benchmark. For Deaths, relative scores were more frequently above 1, with the most substantial deteriorations relative to the benchmark also occurring for this series. For Cases, relative scores tended to stay close to 1, indicating no clear favourite. Differences were overall small between the weighted and unweighted approaches, with a slight preference for the weighted ensembles. Results for all horizons are shown in Fig F in S1 Text, showing that scores tended to worsen slightly when additionally including forecast horizons of 3 and 4 weeks. The clearest exception to the absence of consistent improvement was the Cases series in Poland, particularly under weighted median aggregation.

Fig G in S1 Text shows WIS value spreads decreasing when more models were selected into the ensemble, suggesting ensemble size, rather than selecting for better component models, to be the more important factor when aiming to stabilise ensemble performance. A supplementary analysis extending the selection procedure to additional countries with smaller model pools (Figs H and I in S1 Text) yielded broadly consistent but more unfavourable results. As in the main analysis, no method consistently outperformed the benchmark, thus further reinforcing the importance of ensemble size over selection strategy.

To summarise, improvements in performance were moderate at best, and were not consistent for any aggregation method across all locations and both target series, and substantial advantages over the benchmark were rare.

## Discussion

Our analysis of the number of models contained in an ensemble showed that including more models both on average improved and stabilised aggregate ensemble performance. These improvements however tended to taper off or become more gradual after including 3–4 models. We further found no strong connection between an ensemble’s diversity and its performance. This was the case both when assessing diversity numerically and when doing so qualitatively in terms of classifying modelling approaches. This is in contrast to theoretical accounts of ensemble performance, such as the error-ambiguity decomposition [22], which predict that greater disagreement among accurate component models should reduce ensemble error. At least for the model types and sample sizes available here, that benefit did not materialise.

While results showed that, even with a low number of component models, some recombined ensembles performed better than an ensemble that included all available models, the results of the selection ensemble analysis suggest that it remains unclear how these sets of models could be identified in (pseudo-)real time. In fact, there were some location-target combinations where (almost) none of the smaller ensembles managed to outperform the full Hub ensemble, making the latter the more stable choice. The reduction in performance spread further suggested that there may be less need for model selection once an ensemble includes a moderate number of component models (approximately 4–6 models in our setting).

In particular, the second part of the analysis has shown that selecting for recent better performers did not consistently improve performance and only rarely gave a substantial advantage over a larger equally weighted median ensemble. This might be due to non-stationarity of model performance, making past performance of a model an unreliable predictor of current or future performance. The one exception, Cases in Poland under weighted median aggregation, showed no distinctive pattern in model selection or weighting to set it apart from the other locations, and may reflect sample variation. On the other hand, more sophisticated aggregation methods, such as those that directly optimise component weights with respect to past performance, might leverage individual contributions more successfully than our attempts to select models on a binary basis and additionally weighting by inverse past scores.

Our findings may thus represent an instance of the “forecast combination puzzle” long noted in the economic forecasting literature [2,23,24]: across many forecasting domains, equally weighted combinations match or outperform weighted ones [25], even though statistical theory suggests that optimal performance-based weighting should be achievable. A common explanation is estimation error, since the weights are noisy estimates from finite data [14].

Previous analyses of multi-model infectious disease forecasting efforts have obtained similar results to ours. Fox et al. [10] identified a stabilising effect of larger ensembles, while also reporting diminishing returns in performance for more than 4 models. In a nowcasting context, Amaral et al. [26] did not find consistent improvements from selection and weighting schemes compared to a full unweighted ensemble, while similarly reporting a stabilisation effect of larger ensembles with diminishing returns. Ray et al. [16] employed a more sophisticated weighting scheme than that used in the context of this paper, and found improvements for only one of the two considered data series, COVID-19 Deaths in the United States.

While most of these existing analyses were limited to a single location, our work extends to several locations and two target series, in a setting characterised by intermittent submission and, at times, a small number of available models.

We note several limitations in our analyses. The findings were primarily based on COVID-19 forecasts from 5 European countries over a 12-month period, and generalisation to other diseases, settings, or pandemic phases requires further investigation. Moreover, while we focused on the 5 countries with the highest participation, the underlying model pool remained small and was assembled opportunistically through voluntary contributions. Our results on diversity and model selection hold only across the observed range, which included neither near-identical nor clearly poor models. We thus cannot rule out that including very similar models would have an adverse effect on performance, or that model selection could help when models exist that perform poorly over the entire study period. Furthermore, a larger and more systematically constructed sample, where all models would also be available for all forecast dates, would support deeper analyses, for example by extending the recombination experiment to larger values of *k*. In the recombination experiment, the ensemble diversity analysis generally relied on small samples for the homogeneous group in particular, limiting the strength of conclusions about model-type diversity.

Specific to the selection ensemble, the varying size of the available model pool meant that selecting the *k* best performers carried a different meaning depending on how many models were available: choosing the 5 best from 15 is not equivalent to choosing the 5 best from 6. Furthermore, although the selection ensemble was framed as a real-time procedure, the analysis remained retrospective and thus only simulated real-time performance. A further, empirical limitation is that model dropout was not entirely random with respect to past performance: for Deaths, models with worse recent performance were slightly more likely to drop out the following week (Fig J in S1 Text). This in principle affects which models are available for selection, though the direction of any resulting effect on ensemble performance is unclear.

Extensions of our selection analysis could include the application of alternative weighting schemes, or selection procedures that focus on other aspects of component models, such as a stable submission history. Likewise, a selection scheme could focus on particularly well-performing subgroups of models rather than individual models, as in [10]. Furthermore, it may be valuable to restrict attention to periods of stable model composition and apply a leave-one-out scheme to isolate the contribution of individual models, which would be better suited to a larger and more consistently populated model pool than was available here. Alternative approaches to assessing model diversity could also be explored: our analysis classified models by methodological type and complemented this with a numerical distance measure between predictions, but there might exist more meaningful delineations of model diversity. These could pertain to other modelling choices, such as the data inputs used or whether interventions are explicitly modelled, or directly to forecast behaviour such as the similarity of error patterns across forecast dates.

Taken together, the two parts of our analysis point in the same direction: ensemble performance is shaped more by the number of contributing models than by the selection or weighting of particular ones. A benefit of including more models was apparent in both parts, whereas the composition factors we examined, that is, model diversity in the recombination experiment, and selection and inverse-score weighting in the selection experiment, yielded no consistent advantage. In the absence of consistently better performing component models and/or evidence of better-suited aggregation methods, the practical advice for organisers of multi-model prediction platforms thus remains to collate a moderate number of models (around 4–6) and aggregate them indiscriminately via the unweighted median function.

## Supporting information

S1 Text Supplementary figures. Additional figures on the data, ensemble construction, robustness checks for the recombined and selection ensembles, and submission behaviour by prior performance.
(PDF)
