# Supplementary material for: The influence of ensemble size and composition on the performance of combined real-time COVID-19 forecasts
Source: PLoS Comput Biol. 2026 Nov 3;22(11):e1014908. doi: 10.1371/journal.pcbi.1014908 (PMC13759069; doi:10.1371/journal.pcbi.1014908)
Supplement: Supplementary file 1 [file pcbi.1014908.s001.pdf]

Supporting figures to article submission: The influence of ensemble size and composition on the performance of combined real-time COVID-19 forecasts

Friederike Becker[1*], Katharine Sherratt[2,3], Nikos I. Bosse[2,3], Sebastian Funk[2,3]

1 Institute of Statistics, Karlsruhe Institute of Technology, Karlsruhe, Germany

2 Centre for Mathematical Modelling of Infectious Diseases, London School of Hygiene & Tropical Medicine, London, United Kingdom

3 Department of Infectious Disease Epidemiology and Dynamics, London School of Hygiene & Tropical Medicine, London, United Kingdom

* friederike.becker@kit.edu

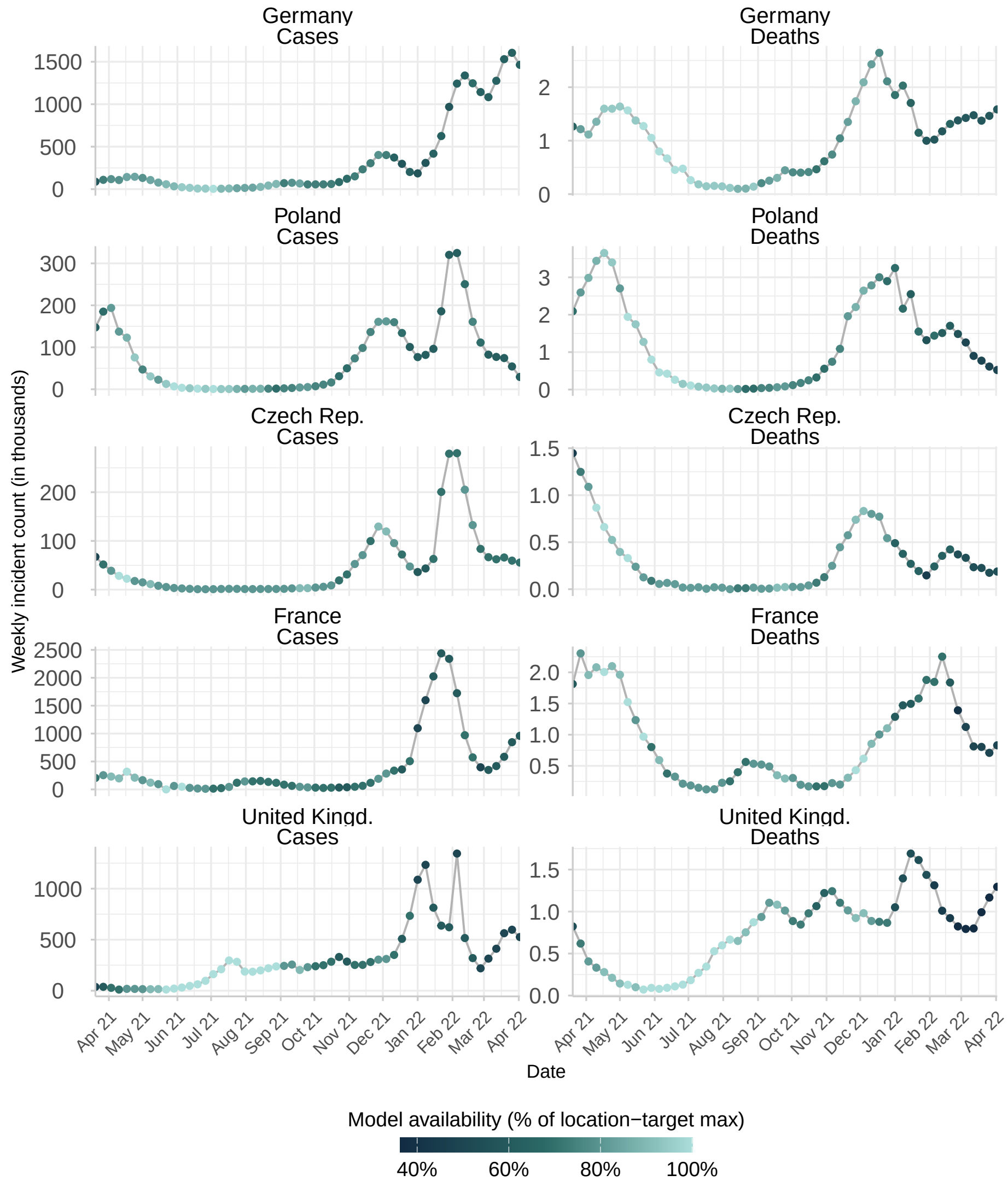

Fig A. Weekly incident COVID-19 Cases and Deaths for the five study locations, between mid-March 2021 and mid-March 2022. Points are coloured by the number of component models available at the corresponding forecast date, expressed as a percentage of the maximum number of models available for that combination of location and target series. Absolute numbers of component models are shown in Fig 1. Note the differing y-axis scales across panels.

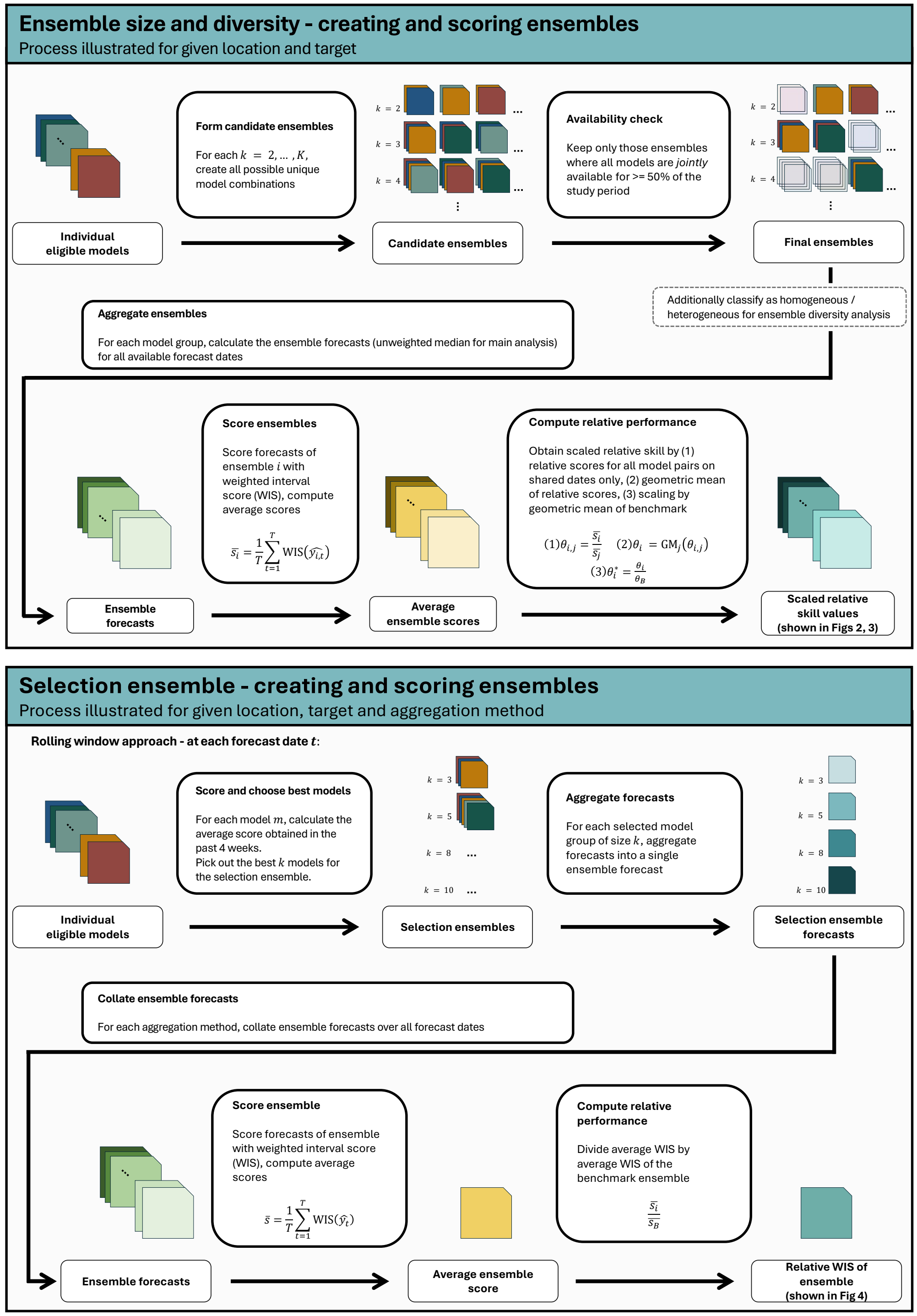

Fig B. Overview of ensemble construction and scoring for the two analyses. Both processes are shown for a single location and target series, and were run separately for each combination. Recombination (top panel) yields many ensembles per k, whereas selection (bottom panel) yields one per k at each forecast date — hence the different approaches to obtaining comparable scores. Results are shown in Figs 2 and 3 (top panel) and Fig 4 (bottom panel).

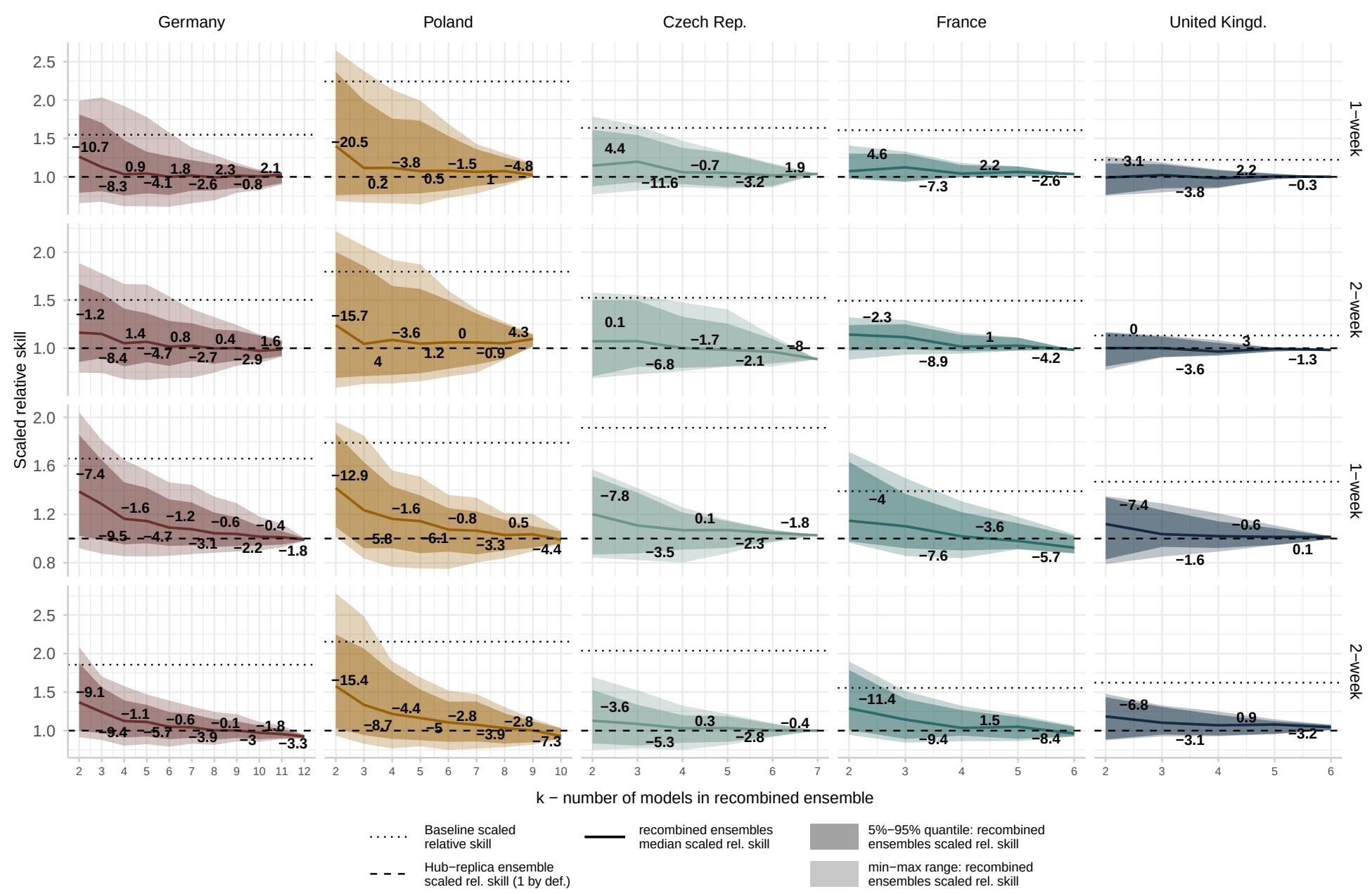

Fig C. Same as Fig 2 in main text, with additionally indicated percentage changes of median scaled relative skill across all recombined ensembles. For instance, a value of -9 shown between $k = 2$ and $k = 3$ indicates a decrease of 9% for median scaled relative skill between $k = 2$ and $k = 3$.

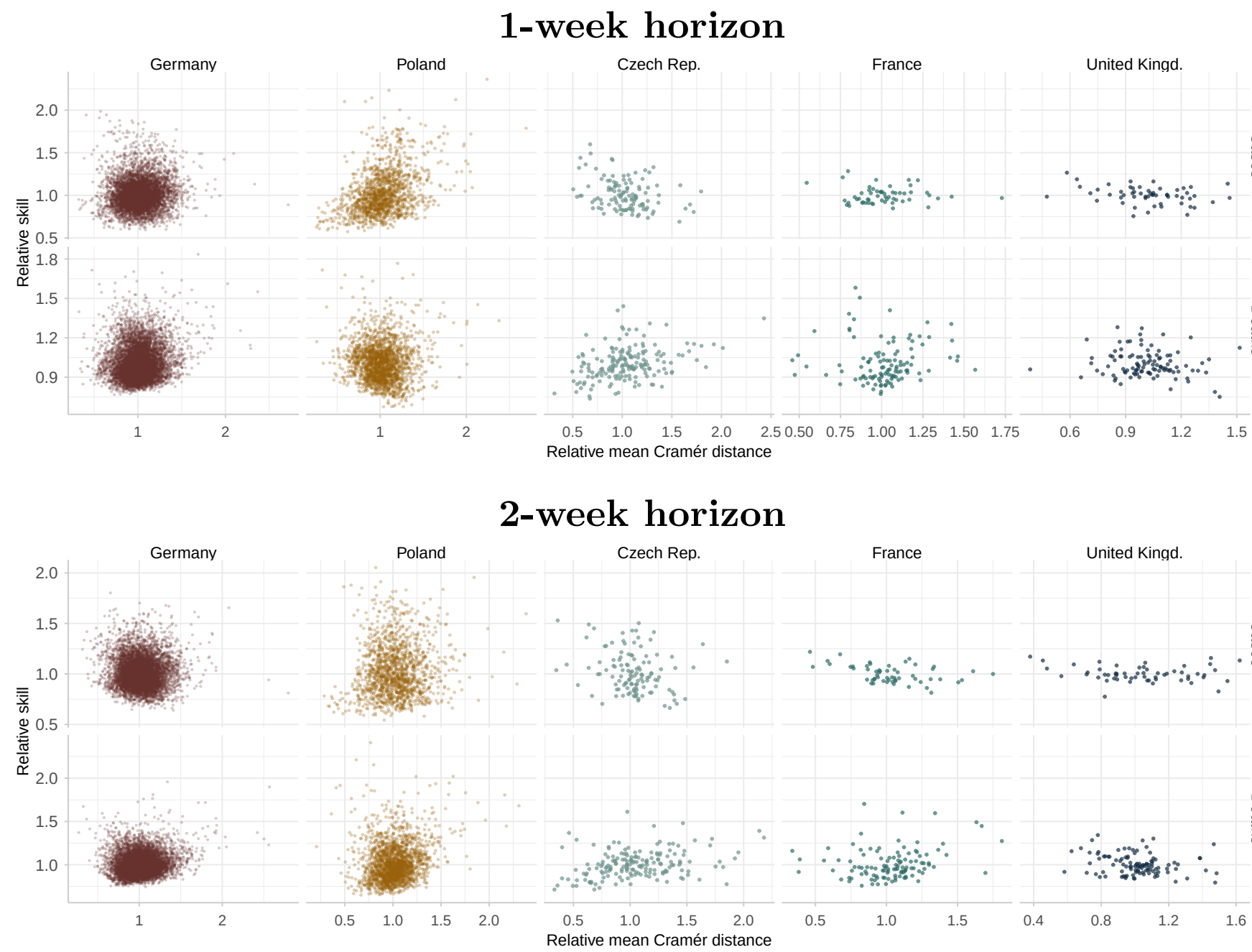

Fig D. Scaled relative skill, plotted against relative mean pairwise Cramér distance, for all recombined ensembles. The top panel shows the 1-week-ahead horizon and the bottom the 2-week-ahead horizon. The Cramér distance is a measure of dissimilarity between probability distributions, see e.g. [1] for a quantile-based representation. Each point represents one recombined ensemble. Note that points' size and alpha values (translucency of points) are slightly adjusted for locations with larger numbers of recombined ensembles (Poland and Germany) to improve legibility.

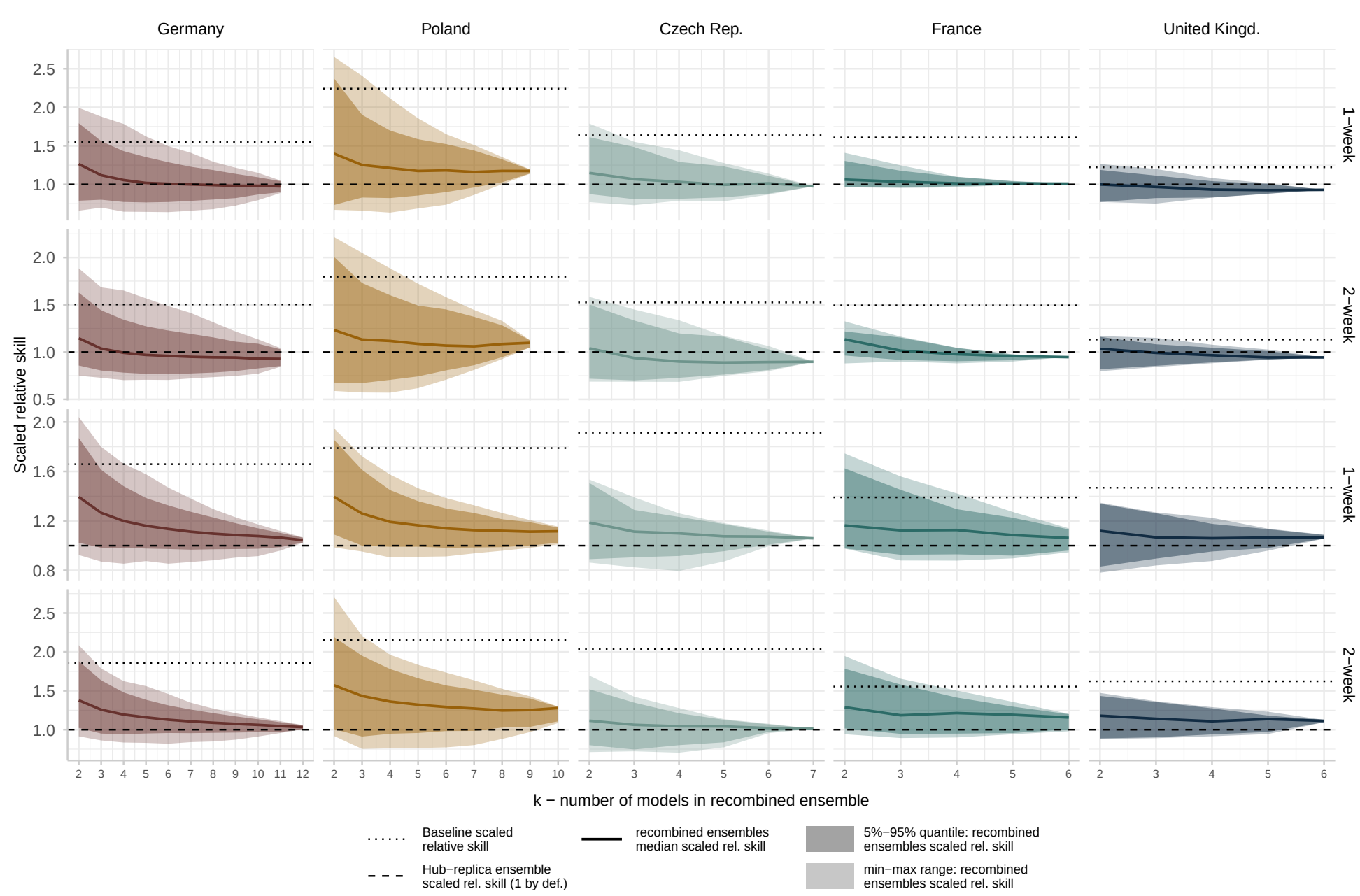

Fig E. Same as Fig 2 in main text, but using the mean function to aggregate recombined ensembles.

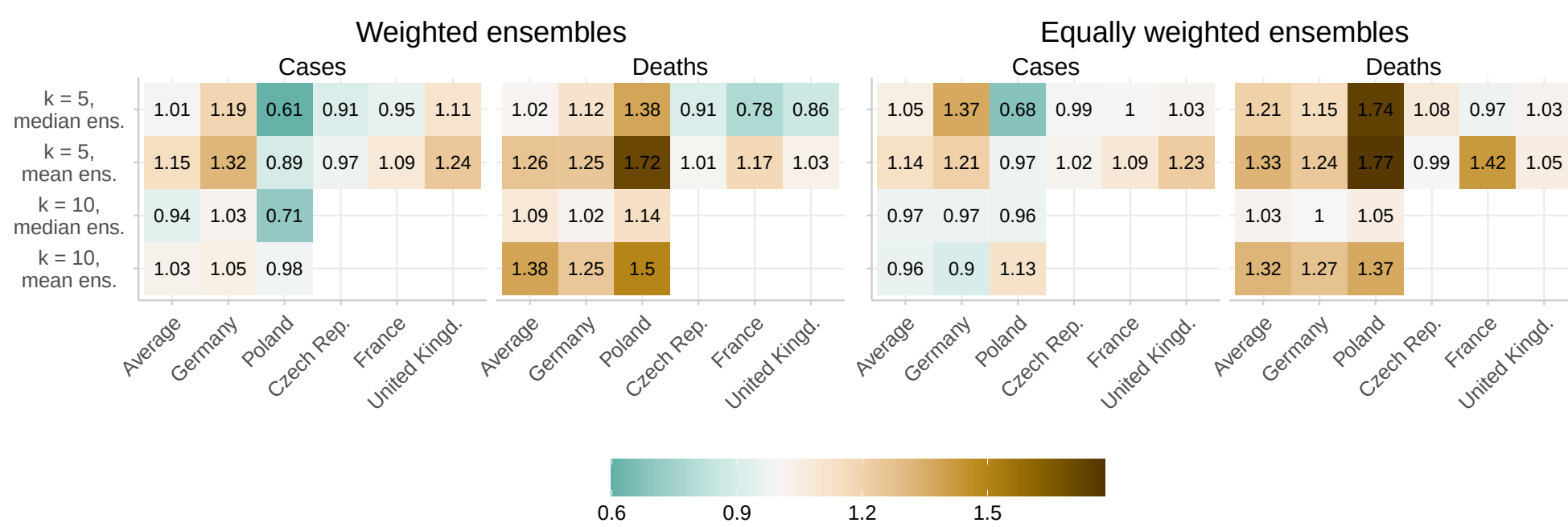

Fig F. Same as Fig 4 in main text, but for all forecast horizons. Note the change in colour scale to accommodate a larger range of relative scores.

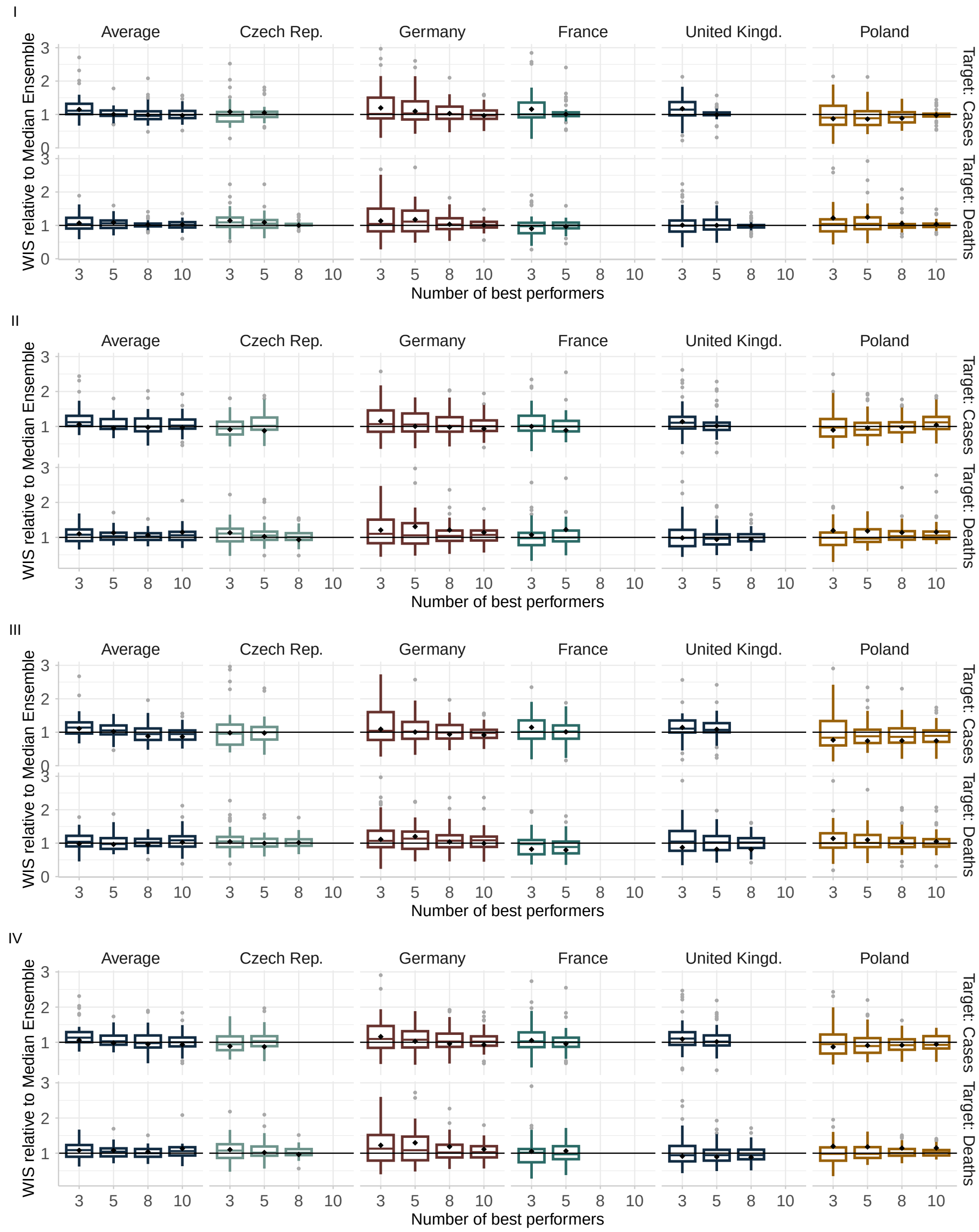

Fig G. WIS spread of the selection ensemble methods relative to the Hub-replica ensemble, by location, target series, and number of best recent performers (k) included. The four panels show the different aggregation methods. (I): equally weighted median. (II): equally weighted mean. (III): inverse score weighted median. (IV): inverse score weighted mean. Outliers with relative scores over 3 were excluded from the plots for legibility - to compensate for this, the diamond-shaped black points show the mean relative score value. Values below one mean that the respective method outperformed the benchmark for the given target. Missing boxplots for a given k indicate that the corresponding location and target type combination lacked a sufficient model pool to support the analysis.

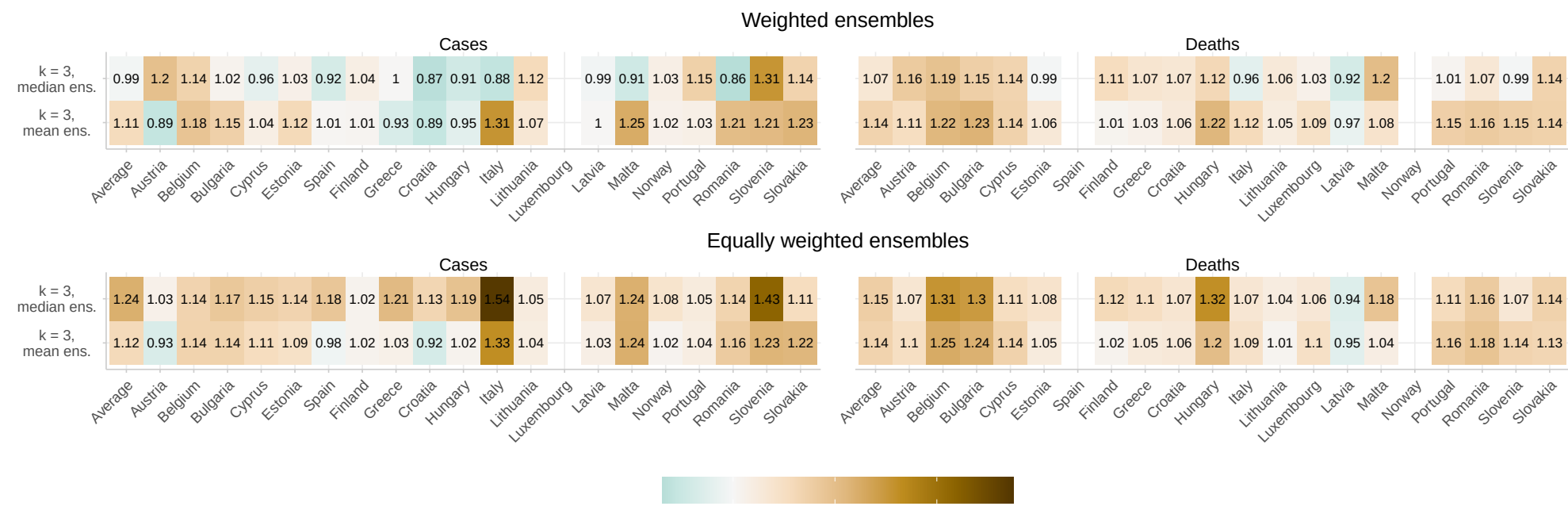

Fig H. Same as Fig 4 in main text, but for $k = 3$ and all remaining eligible countries. Note the change in colour scale to accommodate a larger range of relative scores.

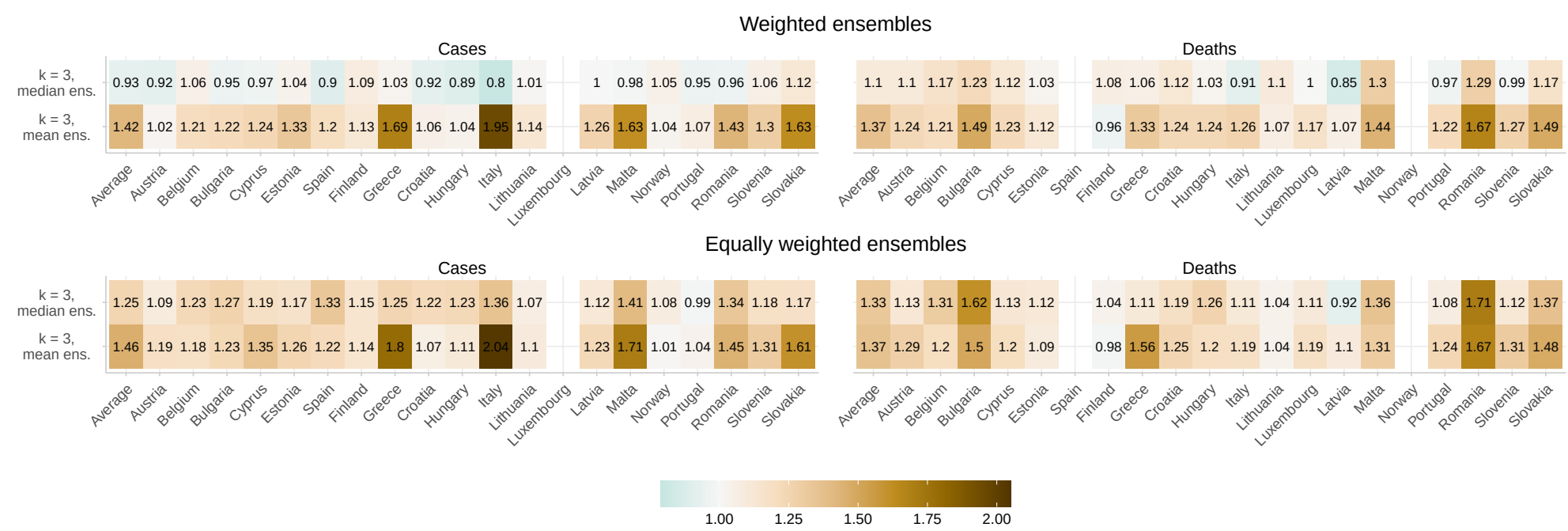

Fig I. Same as Fig 4 in main text, but for $k = 3$, all forecast horizons and all remaining eligible countries. Note the change in colour scale to accommodate a larger range of relative scores.

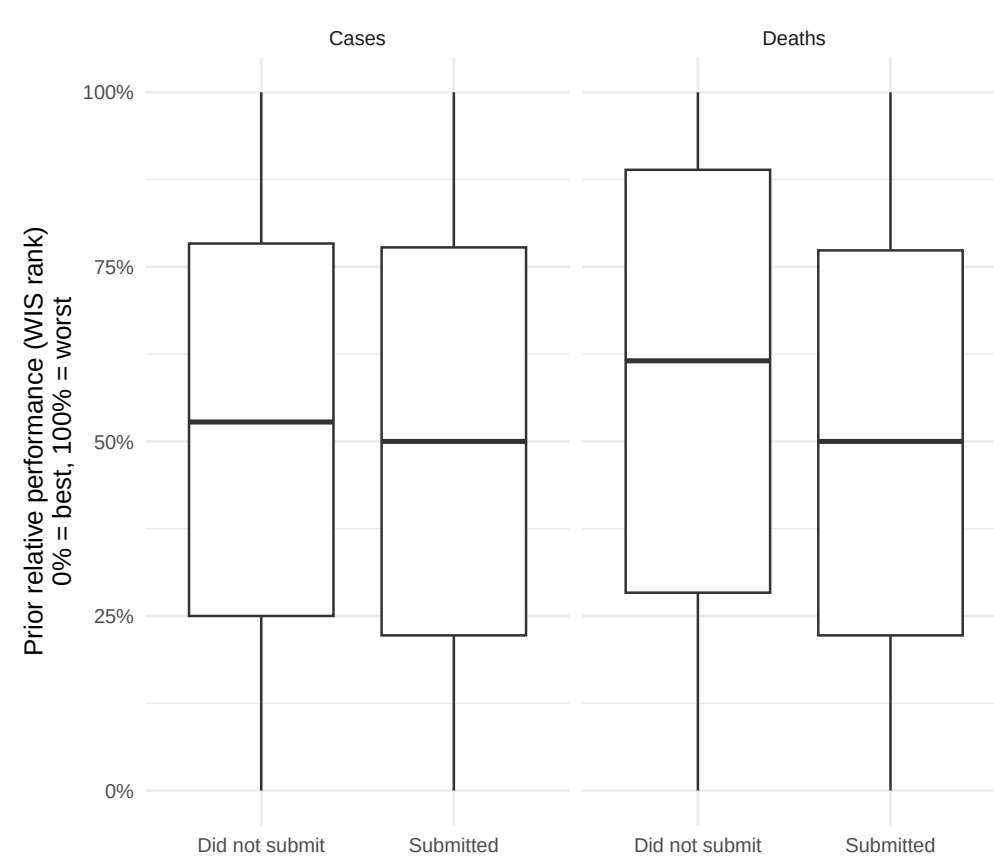

Fig J. Comparison of prior forecasting performance between models that did and did not submit a forecast in a given week. For each location-target-date group, models are ranked by their previous-week 1-week-ahead WIS, the only horizon for which ground truth has realised at the time next submissions are due. 0% indicates the best-performing model and 100% the worst. A shift toward higher ranks for non-submitters indicates that models with worse recent performance were more likely to drop out.

References

1. Resin J, Wolffram D, Bracher J, Dimitriadis T. Shift-Dispersion Decompositions of Wasserstein and Cramér Distances; 2024. Preprint, https://arxiv.org/abs/2408.09770.
