# Supplementary material for: The influence of ensemble size and composition on the performance of combined real-time COVID-19 forecasts
Source: PLoS Comput Biol. 2026 Nov 3;22(11):e1014908. doi: 10.1371/journal.pcbi.1014908 (PMC13759069; doi:10.1371/journal.pcbi.1014908)
Supplement: Supplementary file 2 [file pcbi.1014908.s004.pdf]

PCOMPBIOL-D-26-00122: Point-by-point response to review comments

2nd September 2026

We would like to thank the review team for the constructive reports and thoughtful comments on our paper. In what follows we respond to the referees' individual comments. Throughout, the referees' comments are shown in italics, with our responses in regular text beneath each comment. Changes made independently of the referees' comments are reported in section 'Further changes' at the end of this document.

In preparing this response letter, we used Claude (Anthropic) to help draft and refine our replies. All responses reflect the authors' own assessments.

Please see the file 'diff.pdf' for a detailed listing of the revisions made in comparison to the originally submitted manuscript.

Response to Referee 1

1. Referee comment: *Is it possible to somehow see the time series data in FIG 1 or somewhere. It appears to me that the number of component models is linked to the stage of the epidemic (i.e. teams start to not submit nearing the end and there is some startup delay for some groups). Also is there any possibility that this biases the results?*

   We have included a figure in the supplement showing the time series data alongside model availability (S1 Fig), and added a pointer to the Scope section: "The temporal pattern of model availability alongside epidemic incidence is further illustrated in S1 Fig".
   It is correct that the number of component models varied systematically over the study period: availability was lowest at the start, and also declined during the large winter wave of early 2022, when incidence was highest. As lower incidence periods tend to produce lower absolute scores [1], the scoring procedures used in the paper are chosen to mitigate bias that this would create in the end results:

- For the recombined ensembles, which are the basis for the analysis of ensemble size and diversity, we use pairwise comparisons to score performance. This way of scoring avoids assigning more favourable scores to ensembles on the basis of being available only during low-incidence months. The central assumption underlying this approach, as in [2], is that performing well relative to the set of comparators (here: the other recombined ensembles) is similarly difficult across forecast dates. We have changed the phrasing in the Forecast evaluation section to clarify that our use of pairwise comparisons mitigates this temporal availability bias: "Because absolute WIS values tend to be lower during low-incidence periods, ensembles that happened to be evaluated predominantly during such periods would receive lower average scores."
- The selection ensembles are available for all forecast dates and can be compared directly against the full equally weighted median ensemble. Here the fluctuating model pool raises a conceptual rather than a scoring issue: selecting the k best performers means something different depending on how many models are available, as choosing the 5 best out of 15 is not equivalent to choosing the 5 best out of 6. We have included a paragraph in the discussion acknowledging this limitation: "Specific to the selection ensemble, the varying size of the available model pool meant that selecting the k best performers carried a different meaning depending on how many models were available: choosing the 5 best from 15 is not equivalent to choosing the 5 best from 6."

2. Referee comment: *For the improvement noticed in Poland is there any indication of why this is the case? (or any hint). For example, Poland may have more difference in component performance. I suspect there is no clear indication of why.*

   The hypothesis that greater persistent differences in component performance make past performance a more reliable predictor in Poland is plausible, but examining individual model selection counts and weights revealed no pattern differentiating Poland from the other locations. We have added a sentence to the Discussion noting this, and that the exception is in keeping with the sample variation already highlighted as a key factor: "The one exception, Cases in Poland under weighted-median aggregation, showed no distinctive pattern in model selection or weighting to set it apart from the other locations, and may reflect sample variation."

Response to Referee 2

1. Referee comment: *The authors specifically select time points and countries with the greatest number of model submissions, but it is unclear how a smaller pool of submissions affects ensemble composition and performance. For countries with fewer submissions, how does their composition compare? While more models have been shown to improve ensemble performance, the practical question of how to optimally use a limited set of submissions at any given time point is not addressed.*

The question of how best to use a limited number of submissions is practically relevant, as many European COVID-19 Forecast Hub countries received substantially fewer model contributions than the five countries considered in the main analysis. To address this, we fit the selection ensembles with $k = 3$ (the maximum feasible ensemble size for this group) on all remaining eligible countries, that is, those with a minimum of four models available at every forecast date. Because of the low number of submissions, these countries cannot support the recombination analysis of ensemble size and diversity, so we restricted this extension to the selection experiment. The results are included in the supplementary material (S8 Fig and S9 Fig).

The findings are broadly consistent with those of the main analysis. No selection method consistently outperformed the full equally-weighted median benchmark: the weighted median for Cases came closest, achieving an average relative WIS of 0.99 across locations for the 1- and 2-week horizon (S8 Fig), which cannot be deemed a clear improvement over the benchmark full Hub ensemble. The remaining methods averaged between 1.11 and 1.24 for Cases, while for Deaths all four methods averaged between 1.07 and 1.15. Results were overall more unfavourable for the selection ensemble once the 3- and 4-week horizons were included (S9 Fig). Here, the weighted median averaged at a slightly better but not substantially improved score (0.93) for Cases. The remaining methods ranged from 1.25 to 1.46 for Cases, while for Deaths all methods averaged between 1.10 and 1.37.

We have added a brief summary of this supplementary analysis to the Results section of the manuscript: "A supplementary analysis extending the selection procedure to additional countries with smaller model pools (S8 Fig and S9 Fig) yielded broadly consistent but more unfavourable results. As in the main analysis, no method consistently outperformed the benchmark, thus further reinforcing the importance of ensemble size over selection strategy."

2. Referee comment: *There is no discussion of how missing data are handled or how missingness might influence the findings. It is possible that missing submissions are not missing at random. For example, a modelling team may choose not to submit in a week where their model is performing poorly, meaning that missingness itself selects for better-performing models. The authors should examine whether there is a systematic pattern to the missing data, for instance, whether models that performed poorly in one week were less likely to submit the following week.*

   To examine whether missingness was random with respect to past performance, we compared models' submission status (submit vs. did not submit), in terms of their WIS quantile rank on the most recent 1-week-ahead forecast, that is, the only horizon for which ground truth has realised at the time next submissions are due. The analysis is included in the supplement (S10 Fig). The effect was negligible for Cases, but slightly more pronounced for Deaths, indicating that models with worse recent performance were slightly more likely to drop out the following week. The selection ensemble could in principle be affected by this: a model with a poor one-week-prior score might

drop out even though its four-week-average rank could still have placed it in the top-k selection pool. However, the direction of this effect on selection-ensemble performance is unclear, as it depends on whether the "missed" models would have been beneficial if selected, which we cannot determine from the available data. We have added a note on this to the Limitations: “A further, empirical limitation is that model dropout was not entirely random with respect to past performance: for Deaths, models with worse recent performance were slightly more likely to drop out the following week (S10 Fig). This in principle affects which models are available for selection, though the direction of any resulting effect on ensemble performance is unclear."

Other patterns of missingness are inherent to collaborative forecasting projects: models not being available for the whole study period because teams dropped in or out, or models being available for only some location-target combinations. The scoring procedures used in our analysis, that is, running all analyses separately by location and target, and using pairwise comparisons where necessary, are chosen to address these forms of missingness. We acknowledge, however, that a larger and more systematically constructed dataset would enable analyses we cannot conduct here, and have included a note on this in the Limitations: “A larger and more systematically constructed sample, where all models would also be available for all forecast dates, would support deeper analyses, for example by extending the recombination experiment to larger values of k."

3. Referee comment: *The rationale for examining model composition changes at each time point is unclear. In practice, is it realistic or useful for the models contributing to an ensemble to change continuously? This approach also complicates comparison, as each time point reflects a different composition. An alternative approach worth considering would be to identify periods of stable model composition and apply a leave-one-out or similar method to assess different ensemble configurations. The authors should also consider whether examining the best-performing ensemble across the full time period would be informative.*

   The selection ensemble is a proposal for how to best construct an ensemble in real-time, based on information on component model performance as it becomes available. With it, we investigate whether an ensembling scheme of selecting models that have performed well in the recent past can better leverage contributions from individual forecasters. Continuously updating ensemble composition is not only realistic in this setting but reflects the initial hypothesis that with access to recent performance data it might be beneficial to update model selection from week to week. The specific design aims to reflect conditions as observed in the European Forecast Hub, that is, a limited number of forecasts and intermittent submission.
   We agree that the best-performing ensemble across the full time period is informative as a performance ceiling, and the distribution of performance across all recombined ensembles in the first part of our analysis provides this information about the range of achievable performance. However, the optimal ensemble necessarily can only be identified in hindsight and cannot guide real-time decision making, which is the setting

we aim to address. Restricting attention to periods of stable model composition and applying a leave-one-out (or similar) scheme would isolate the contribution of individual models without the confounding effect of a changing model pool. We agree this is worth pursuing. In the European Hub data, however, stretches of stable composition are short and infrequent (Fig 1), which limits what such an analysis could reveal here; it would be better suited to a larger and more consistently populated model pool. We note this in the Discussion: "Furthermore, it may be valuable to restrict attention to periods of stable model composition and apply a leave-one-out scheme to isolate the contribution of individual models, which would be better suited to a larger and more consistently populated model pool than was available here."

4. Referee comment: *The discussion does not adequately address the study's limitations, directions for future work, or how the findings situate within the broader literature. Additionally, rather than directing the reader to another paper for context on the "forecast combination puzzle," the authors should provide a brief summary within the manuscript itself.*

   We have expanded the Discussion to address the points raised. We added a dedicated limitations paragraph, covering the scope of the analysis, the opportunistic and limited nature of the underlying model pool, small sample sizes in the ensemble diversity analysis, the inconsistency arising in the selection ensemble because of a varying model pool size, the retrospective framing, and the non-random missingness pattern identified in response to comment 2. We further added a note that a larger and more systematic sample of models would support deeper investigation. We have expanded the "Extensions" paragraph with several further directions: selection procedures based on other model characteristics, such as a stable submission history; selection over well-performing subgroups of models rather than individual ones; a leave-one-out scheme applied to periods of stable model composition; and alternative ways of characterising model diversity, e.g., by data inputs used, treatment of interventions, or the similarity of error patterns across forecast dates. We have further expanded the Discussion to situate our findings within the broader forecast-combination literature beyond infectious-disease forecasting. We added references to the classical work on combining forecasts and to the error-ambiguity account of ensemble diversity, together with the in-text summary of the "forecast combination puzzle".

5. Referee comment: *The manuscript would benefit from greater narrative cohesion. The sections feel somewhat disconnected, and clearer transitions and a more unified storyline would strengthen the overall presentation.*

   We appreciate the referee's suggestion and agree that the narrative can be made more cohesive. The two analyses do address methodologically distinct questions, that is, the recombination experiment examines how ensemble size and diversity affect performance, while the selection ensemble investigates whether past performance can guide model selection in real time; furthermore, they use different evaluation approaches (pairwise comparisons versus direct WIS ratios). We acknowledge that this can make

the two strands appear disconnected.
To address this, we have added paragraphs in key places aiming to strengthen a joint framing and emphasize the analyses' complementary nature:

- In concluding the introduction, we added the following paragraph: "We approach this from two complementary angles. First, we systematically vary ensemble size and composition through a recombination procedure, investigating how performance depends on the number and diversity of contributing models when these are sampled from the available pool. Second, we investigate if ensembles that actively select for recent better-performing models can improve upon an equally-weighted aggregation of all available models. Together, these analyses examine whether ensemble performance is driven more by the size of the contributing pool or by its composition in terms of model diversity and individual performance."
- To better bridge the two analyses in the results section we added the following sentence to the beginning of the selection ensemble results: "While the recombination experiment showed that more models generally improved and stabilised performance, it left open whether a full ensemble could be outperformed by deliberately choosing models that performed well in the recent past, which we investigate next."
- In the discussion, we commence the concluding paragraph with a statement aiming to unify the conclusions from the analyses: "Taken together, the two parts of our analysis point in the same direction: ensemble performance is shaped more by the number of contributing models than by the selection or weighting of particular ones. A benefit of including more models was apparent in both parts, whereas the composition factors we examined, that is, model diversity in the recombination experiment, and selection and inverse-score weighting in the selection experiment, yielded no consistent advantage."

Furthermore, we have reordered the Methods section for improved logical flow (see our response to Other comments 2c) and included a visual representation of all analyses (see our response to Other comments 1).

Other comments

1. Referee comment:*The manuscript could benefit from some visual representation of how individual alternative ensembles were created and compared.*

   We have included a visual representation for the recombination and selection procedures (S2 Fig) and added pointers in the respective sections in the Methods section.

2. Referee comment:*The methods could benefit from reorganisation. In some sections you introduce concepts that are not mentioned until the following sections. This makes the methods difficult to follow. Specifically,*

a) *Page 4, line 59: "we generally compared the performance of alternatively constructed ensembles to that of a "Hub-replica" ensemble,. . . " You haven't yet discussed what alternative ensembles are or how they are constructed.*

b) *Page 5, line 74: "For our study, we only considered "full" forecasts,..." You haven't previously described the scope of forecasts made for the European Forecast Hub, so if a reader is unfamiliar with it, they will be lost. I recommend adding a sentence or two introducing the Hub.*

c) *I think the section "Construction of ensembles of different size" should be moved before "Predictive Performance".*

Regarding point a), we removed the forward reference to "alternatively constructed ensembles" (line 67 in the revised manuscript version), replacing it with a simpler introduction of the Hub-replica ensemble as the benchmark: "In our analysis, we used a "Hub-replica" ensemble as a benchmark for all comparisons."

Regarding point b), the Hub, its scope (locations and targets) and its submission format are introduced in the beginning of the "Scope" section (lines 51 and following). The "full" forecasts paragraph referred to here builds on this submission format. We have additionally clarified at the relevant point that the choice to restrict to full forecasts follows the Hub's own eligibility criteria for inclusion in the official ensemble (see also our response to minor comment 8).

Regarding point c), we thank the referee for this suggestion and agree. We have moved both construction sections ("Construction of ensembles of different size" and "Construction of selection ensembles") ahead of the evaluation section, so that the reader is introduced to how the alternative ensembles were built before encountering how they were scored. We have also renamed the former "Predictive performance" section to "Forecast evaluation", which we feel more accurately describes its content.

3. Referee comment: *Page 13, lines 232-239: If the optimal selection of models cannot be determined in real time, what is the justification for the approach taken here? The paper aims to guide practitioners in the use of ensemble modelling frameworks, yet this paragraph undermines that goal by conceding that the chosen method of combining models may have limited real-time utility. The authors should clarify how these findings are intended to be applied in practice.*

   The sentence was intended to connect results from the recombination experiment with those from the selection ensemble: having tested whether past performance can guide real-time model selection, and found it does not consistently do so, we were aiming to conclude that identifying well-performing model subsets in real time remains an open problem. We agree the original phrasing was ambiguous and have revised it to say "..., the results of the selection ensemble analysis suggest that it remains unclear how these sets of models could be identified in (pseudo-)real time.", making the intended logical connection more explicit.

Minor questions

1. Referee comment: *Page 3, lines 24-28: Expand on this paragraph, it's relevance is not clear.*

   We have revised this paragraph, which introduces the analysis of ensemble diversity, in two ways. First, we tightened the language and added a concluding sentence making explicit both the gap in the existing literature and how the present work addresses it: "It thus remains unclear whether diversely composed ensembles outperform those of a single modelling type, a question we address directly." Second, we reordered the list of contributing factors in the preceding paragraph to match the order in which they are subsequently addressed in the text, moving diversity before individual predictive performance.

2. Referee comment: *Page 4, line 36: define "these factors"*

   We incorporated the concrete list of factors into the relevant sentence: "In this work we explore the impact of these factors, that is, ensemble size, component model diversity, and individual model performance, in the context of the European COVID-19 Forecast Hub...". We accordingly removed the following sentence to avoid duplication.

3. Referee comment: *Page 4, line 39: how can you assess diversity if models are all opportunistically included?*

   Ensemble diversity is assessed retrospectively through the recombination approach described in the Methods: all possible ensembles of size k are constructed from the available model pool and systematically classified as either "homogeneous" (that is, less diverse) or "heterogeneous" (that is, more diverse) depending on whether they consist of one or multiple modelling types. This allows a systematic comparison of diverse versus non-diverse ensembles within an opportunistically assembled model base.

4. Referee comment: *Page 4, line 50: Is the data for all countries considered to be the "ground truth"? How can you be sure that the data for all countries was of the same quality? Also, why were these countries selected?*

   We used truth data from Johns Hopkins University (JHU), used by the official European COVID-19 Forecast Hub and all comparable analyses of both the European and US hubs [2, 3, 4]. We acknowledge that reporting standards and data quality likely varied across countries; however, using a single standardised data source that is consistent with the official Hub and with prior analyses allows for meaningful comparison across studies.
   Country selection was based on the number of available models per country: constructing alternative ensembles along the three axes of size, diversity, and individual performance was only feasible at meaningful sample sizes for the countries with the highest participation, namely Germany, Poland, the Czech Republic, the United Kingdom and France. This rationale is described in the Methods section in lines 75 and following.

5. Referee comment: *Page 4, line 59: "we generally compared. . . " when was this not the comparator?*

   We removed the word "generally". The Hub-replica ensemble was used as the benchmark throughout, making this word choice unnecessarily ambiguous.

6. Referee comment: *Page 5, line 64: "size of the available model base" Do you mean the number of models in the ensemble?*

   We replaced "model base" with "model pool" to make clear that this refers to the total set of available models from which alternative ensembles were constructed, as opposed to the number of models in any given ensemble (denoted k).

7. Referee comment: *Page 5, line 68: so participants can choose which countries they contribute to? Would this affect the model selection to choose from?*

   Yes, participation was voluntary and modelling teams could choose which countries to submit forecasts for. This does affect the available model pool, which consequently varies by location and target, as shown in Fig 1. We account for this directly by running all analyses separately for each location-target combination. We have clarified this in the Scope section by including the following paragraph: "Modelling teams were free to choose which combinations of location and target series they submitted forecasts for, leading to substantial variation in model availability across location-target combinations. Since the set of contributing models differed across these combinations, we conducted all analyses separately by location and target."

8. Referee comment: *Page 5, line 74: why did you choose the definition for "full" forecasts?*

   The definition of "full" forecasts follows the Hub's own eligibility criteria for inclusion in the official ensemble. We added a sentence in the paper that clarifies this: "This choice is consistent with the Hub's own eligibility criteria for inclusion in the official ensemble.", with a citation to the archived forecast hub wiki where this is stated.

9. Referee comment: *Page 5, line 83: Why not test ensembles on the same training sample for easier comparison rather than use "distinct subsets of the training sample"?*

   Due to the intermittent model submission, different recombined ensembles are available for different subsets of forecast dates. Restricting to a common set of dates would substantially reduce the dataset, and would do so in a non-representative way, because only periods of uniformly high participation, which tend to coincide with specific phases of the pandemic, would be retained. The approach to score alternative ensembles with pairwise comparisons addresses this directly, enabling fair comparison across ensembles with partially overlapping availability without discarding data.
   To make this reasoning more clear in the text, we changed the formulation "In the first part of our analysis, we constructed alternative ensembles that each made forecasts on distinct subsets of the training sample" to "In the first part of our analysis (see section 'Construction of ensembles of different size'), different ensembles were not generally available on the same forecast dates."

10. Referee comment: *Page 6, line 94: "on a rolling basis" Does this mean that the composition of the ensemble changed over time?*

    Yes, at each forecast date, the k best performers from the preceding 4-week window were selected, meaning the ensemble composition could change from week to week. This is intentional, reflecting a real-time setting in which one would attempt to optimise ensemble composition given a limited number of available model submissions, based on those models' most recent performance data.

11. Referee comment: *Page 6, line 108: Does "recombined" = "alternative" or are these different types of ensembles?*

    "Alternative ensemble" is our umbrella term for any ensemble constructed as an alternative to the Hub-replica benchmark; "recombined ensembles" (part 1) and "selection ensembles" (part 2) are its two subtypes. To remove ambiguity in the given part, we changed it to just say "ensembles".

12. Referee comment: *Page 7, lines 123-127: I don't understand the pairwise comparisons. Did you compare each alternative to the comparator?*

    We expanded the description of the pairwise comparison procedure in the Methods section: "Briefly, this procedure computes relative skill for each pair of ensembles as the ratio of their average scores on shared forecast dates only, thus avoiding the bias introduced by incomplete overlap in availability. Each ensemble's total relative score is then computed as the geometric mean of relative skill values across all comparators, and subsequently scaled against the Hub-replica's relative score."
    We also note that the referenced lines 123 to 127, which described a 75% sampling step for Germany due to computational constraints, have been removed from the manuscript: we obtained access to larger computing resources and ran the full recombination set, which did not meaningfully change results (see Section "Further changes" further down).

13. Referee comment: *Page 8, line 148: What is the value cited from [14]?*

    We clarified the text to make the value explicit: [5] used a 3-week window in the cited analysis.

14. Referee comment: *Page 8, line 168: Is the baseline model the carry one forward for this part of the analysis?*

    Yes, the baseline is the same last-value-carried-forward model introduced in the Scope section. To remove the ambiguity at this point in the text, we now refer to it as "the naive baseline model" throughout, instead of just "the baseline model".

15. Referee comment: *Page 13, line 228: "These improvements however tended to taper off or become more gradual after 2 to 4 models." How many were higher than this? 2 models seems low.*

    Upon reflection, we agree that 2 models is an overly optimistic lower bound for where improvements taper off, and is not well supported by our results. We have updated

the Discussion to read "3 to 4 models" to more accurately reflect the range shown in Fig 2.

16. Referee comment: *Page 13, line 240: define "moderate"*

    We added a quantitative clarification in the Discussion and in the Conclusion, defining "moderate" as approximately 4–6 models based on the stabilisation of performance visible in Fig 2.

17. Referee comment: *Page 13, lines 241-242: This conclusion was not clear from the results, so I suggest clarifying the results text.*

    We have lightly revised and extended the Results paragraphs to better support the conclusion drawn in the Discussion. Specifically, we added a clause to the closing summary explicitly stating that "substantial advantages over the benchmark were rare", describe the relative performance in more detail for the two targets, note the clearest exception (Poland) more clearly, and made small adjustments elsewhere for clarity. We hope that the conclusion in the Discussion is now more clearly grounded in the Results text.

18. Referee comment: *Page 14, line 245: "sophisticated aggregation methods" such as?*

    We clarified what is meant by "sophisticated aggregation methods", specifying approaches that directly optimise component weights with respect to past performance, as opposed to the more heuristic inverse-performance weighting used here.

19. Referee comment: *Figure 4: the time horizons should be indicated on the figure. Which rows correspond to which time horizon?*

    The 1- and 2-week horizons are averaged together in Fig 4; the rows correspond to different values of k and aggregation methods. We clarified the caption to read "averaged for the 1- and 2-week forecast horizons" to avoid this confusion.

Further changes

Independently of the referees' comments, we made the following changes to the manuscript.

- We noticed an error in the coding of weighted median aggregation, leading to non-monotonic quantiles in a few instances. We fixed the error, which led to minor changes in aggregate scores. The corresponding updates to Fig 4 are visible in the tracked-changes version of the manuscript.

- We obtained access to larger computing resources and removed the 75% sampling step for scoring the recombined ensembles from Germany (affects Figs 2, 3, S3 and S5, updates are visible in the tracked-changes version of the manuscript). This did not change results in a meaningful way.
  We thus removed all mentions of this step from the manuscript.

- We added a short intuitive explanation of how the WIS evaluates forecast intervals in the Forecast evaluation section.
- We also made minor corrections and clarifications throughout the text to improve readability; none affect the results or conclusions. All changes are highlighted in the file 'diff.pdf'.
- In accordance with PLOS policy on the use of AI, we added a short subsection "Use of AI tools" to the Methods, disclosing our use of AI tools during the revision.

References

[1] Bracher J, Ray E, Gneiting T, Reich N. Evaluating epidemic forecasts in an interval format. PLOS Computational Biology. 2021;17(2):e1008618.

[2] Cramer E, Ray E, Lopez V, Bracher J, Brennen A, Castro Rivadeneira A, et al. Evaluation of individual and ensemble probabilistic forecasts of COVID-19 mortality in the United States. Proceedings of the National Academy of Sciences. 2022;119(15):e2113561119.

[3] Sherratt K, Gruson H, Grah R, Johnson H, Niehus R, Prasse B, et al. Predictive performance of multi-model ensemble forecasts of COVID-19 across European nations. eLife. 2023;12:e81916.

[4] Ray E, Brooks L, Bien J, Biggerstaff M, Bosse N, Bracher J, et al. Comparing trained and untrained probabilistic ensemble forecasts of COVID-19 cases and deaths in the United States. International Journal of Forecasting. 2023;39(3):1366-83.

[5] Bracher J, Wolffram D, Deuschel J, Görgen K, Ketterer J, Ullrich A, et al. A pre-registered short-term forecasting study of COVID-19 in Germany and Poland during the second wave. Nature Communications. 2021;12(1):5173.
