# Supplementary material for: The influence of ensemble size and composition on the performance of combined real-time COVID-19 forecasts
Source: PLoS Comput Biol. 2026 Nov 3;22(11):e1014908. doi: 10.1371/journal.pcbi.1014908 (PMC13759069; doi:10.1371/journal.pcbi.1014908)
Supplement: Supplementary file 3 [file pcbi.1014908.s002.docx]

The central question of this paper: how ensemble model composition can be improved by considering model type and prior performance, is both necessary and useful. However, I have several concerns about the study design and manuscript.

1. The authors specifically select time points and countries with the greatest number of model submissions, but it is unclear how a smaller pool of submissions affects ensemble composition and performance. For countries with fewer submissions, how does their composition compare? While more models have been shown to improve ensemble performance, the practical question of how to optimally use a limited set of submissions at any given time point is not addressed.
2. There is no discussion of how missing data are handled or how missingness might influence the findings. It is possible that missing submissions are not missing at random. For example, a modelling team may choose not to submit in a week where their model is performing poorly, meaning that missingness itself selects for better-performing models. The authors should examine whether there is a systematic pattern to the missing data, for instance, whether models that performed poorly in one week were less likely to submit the following week.
3. The rationale for examining model composition changes at each time point is unclear. In practice, is it realistic or useful for the models contributing to an ensemble to change continuously? This approach also complicates comparison, as each time point reflects a different composition. An alternative approach worth considering would be to identify periods of stable model composition and apply a leave-one-out or similar method to assess different ensemble configurations. The authors should also consider whether examining the best-performing ensemble across the full time period would be informative.
4. The discussion does not adequately address the study's limitations, directions for future work, or how the findings situate within the broader literature. Additionally, rather than directing the reader to another paper for context on the "forecast combination puzzle," the authors should provide a brief summary within the manuscript itself.
5. The manuscript would benefit from greater narrative cohesion. The sections feel somewhat disconnected, and clearer transitions and a more unified storyline would strengthen the overall presentation.

Other comments

1. The manuscript could benefit from some visual representation of how individual alternative ensembles were created and compared.
2. The methods could benefit from reorganisation. In some sections you introduce concepts that are not mentioned until the following sections. This makes the methods difficult to follow. Specifically,
   a. Page 4, line 59: “we generally compared the performance of alternatively constructed ensembles to that of a “Hub-replica” ensemble,…” You haven’t yet discussed what alternative ensembles are or how they are constructed.
   b. Page 5, line 74: “For our study, we only considered “full” forecasts,..” You haven’t previously described the scope of forecasts made for the European Forecast Hub, so if a reader is unfamiliar with it, they will be lost. I recommend adding a sentence or two introducing the Hub.
   c. I think the section “Construction of ensembles of different size” should be moved before “Predictive Performance”.
3. Page 13, lines 232-239: If the optimal selection of models cannot be determined in real time, what is the justification for the approach taken here? The paper aims to guide practitioners in the use of ensemble modelling frameworks, yet this paragraph undermines that goal by conceding that the chosen method of combining models may have limited real-time utility. The authors should clarify how these findings are intended to be applied in practice.

Minor questions

1. Page 3, lines 24-28: Expand on this paragraph, it’s relevance is not clear.
2. Page 4, line 36: define “these factors”
3. Page 4, line 39: how can you assess diversity if models are all opportunistically included?
4. Page 4, line 50: Is the data for all countries considered to be the “ground truth”? How can you be sure that the data for all countries was of the same quality? Also, why were these countries selected?
5. Page 4, line 59: “we *generally* compared…” when was this not the comparator?
6. Page 5, line 64: “size of the available model base” Do you mean the number of models in the ensemble?
7. Page 5, line 68: so participants can choose which countries they contribute to? Would this affect the model selection to choose from?
8. Page 5, line 74: why did you choose the definition for “full” forecasts?
9. Page 5, line 83: Why not test ensembles on the same training sample for easier comparison rather than use “distinct subsets of the training sample”?
10. Page 6, line 94: “on a rolling basis” Does this mean that the composition of the ensemble changed over time?
11. Page 6, line 108: Does “recombined” = “alternative” or are these different types of ensembles?
12. Page 7, lines 123-127: I don’t understand the pairwise comparisons. Did you compare each alternative to the comparator?
13. Page 8, line 148: What is the value cited from [14]?
14. Page 8, line 168: Is the baseline model the carry one forward for this part of the analysis?
15. Page 13, line 228: “These improvements however tended to taper off or become more gradual after 2 to 4 models.” How many were higher than this? 2 models seems low.
16. Page 13, line 240: define “moderate”
17. Page 13, lines 241-242: This conclusion was not clear from the results, so I suggest clarifying the results text.
18. Page 14, line 245: “sophisticated aggregation methods” such as?
19. Figure 4: the time horizons should be indicated on the figure. Which rows correspond to which time horizon?
